# Acute Respiratory Distress Syndrome in Young Postrenal Transplant Patients Receiving Basiliximab

**DOI:** 10.1155/crcc/1272468

**Published:** 2025-02-17

**Authors:** Kalkena Sivanesam, Scott K. Breeden, Stephen Brannan

**Affiliations:** Department of Anesthesiology, University of Nebraska Medical Center, Omaha, Nebraska, USA

## Abstract

Renal transplants have been increasing in number due to the rise in end-stage renal disease (ESRD) worldwide. Transplant is a good approach to management of renal disease, as it offers patients a better quality of life. However, complications such as acute graft rejection remain a concern in all transplant patients. Basiliximab is an antibody commonly used as part of acute rejection prevention in renal transplantation. This antibody has been demonstrated to have comparable efficacy as other agents currently used, with the added benefit of decreasing the amount of steroids required for adequate immunosuppression. The general side effect profile for basiliximab includes infection, gastrointestinal disturbance, hypertension, and hyperkalemia. Respiratory system-related effects include dyspnea and upper respiratory tract infections, most of which have been documented to be mild or moderate in severity. However, a search of the literature reveals that there are a few reported cases of severe respiratory side effects in patients receiving basiliximab after renal transplants. In this report, we discuss two separate cases of acute respiratory distress syndrome (ARDS) that occurred in two young male patients. Both patients were without any other comorbidities, who had recently undergone renal transplantation and received basiliximab as part of acute rejection prevention. Both cases have a similar timeline of symptom onset, and both patients quickly developed severe respiratory failure requiring extracorporeal membrane oxygenation (ECMO) for respiratory support. Analysis of possible causes of respiratory failure points to a common medication that was administered to both patients. This report adds to a growing number of cases that suggest basiliximab may play a role in the development of respiratory failure in young patients undergoing renal transplantation surgery.

## 1. Introduction

Basiliximab is an interleukin-2 (IL-2) receptor antibody derived from recombinant chimeric murine/human IgG1 monoclonal antibodies. Kidney Disease Improving Global Outcomes (KDIGO) guidelines recommend the use of basiliximab as part of the induction phase for acute rejection prevention in renal transplantation [1]. The efficacy is comparable to that of other interleukin receptor antibodies, and the use of basiliximab has been shown to decrease the required dose of other antirejection medications, including steroids, thus reducing the side effect profile of those medications [2].

Basiliximab is independently associated with a similar side effect profile as other IL-2 receptor antibodies as part of triple immunotherapy. Excluding infection, basiliximab's most reported adverse event was related to gastrointestinal disorders. While some upper respiratory tract infections have been reported, acute respiratory distress syndrome (ARDS) or pulmonary edema have not typically been associated with this agent [3]. Literature review showed very few reports that discuss an association between basiliximab and severe respiratory failure. One report published in 2015 describes an incidence of ARDS, shock, and multiple organ failure with cardiac arrest in a 20-year-old patient given basiliximab postrenal transplantation [4]. The patient required extracorporeal membrane oxygenation (ECMO) and continuous hemodialysis post cardiac arrest and eventually recovered. The evaluation of the patient showed no other etiology for these events, leading to the hypothesis that basiliximab was the cause.

Herein, we describe two cases of young adult patients who underwent deceased donor renal transplantation and experienced ARDS requiring ECMO. These cases present evidence of a possible association between basiliximab and ARDS in young patients. Understanding these cases could serve as the basis for further investigation into the association between this monoclonal antibody and pulmonary complications in a high-risk population.

## 2. Case Report

### 2.1. Case 1

A male patient in his early 30s presented for renal transplantation. The patient had end-stage renal disease (ESRD) secondary to congenital obstructive nephropathy and congenital vesicoureteral reflux complicated by hypertension. The patient was on home hemodialysis and determined to be a candidate for renal transplantation. The patient underwent deceased donor renal transplantation successfully. As part of acute organ rejection induction protocol, the patient received 20 mg basiliximab. The patient's postoperative course was unremarkable initially. However, on Postop Day 3, the patient received his second dose of basiliximab 20 mg and soon after began showing signs of respiratory distress. The patient was given supplemental oxygen, and a full workup for potential causes of hypoxic respiratory failure including echocardiogram (ECHO), ventilation/perfusion (V/Q) scan, and computed tomography (CT) scan was initiated. Given the patient's supplemental oxygen requirement steadily increased to more than 15 L, the patient was transferred to the intensive care unit (ICU) for further management. Results from imaging showed a normal ECHO with ejection fraction of 55%–60%. The V/Q scan did not show any evidence of pulmonary embolism. The CT scan showed bilateral consolidative ground glass opacities and interlobular septal thickening. These results could be secondary to edema, hemorrhage, ARDS, or infection.

In the ICU, the patient was started on noninvasive positive pressure ventilation (NIPPV) with signs of improvement in respiratory status. The patient was also noted to have significant hypertension, so nicardipine was started to help manage the blood pressure along with furosemide for diuresis. Despite an initial positive response, the patient's respiratory status continued to deteriorate throughout the day. Eventually, the patient required intubation. During intubation, pink frothy sputum was noted in the airway. The patient continued to struggle with ventilation and oxygenation, so the patient was placed on a high positive end-expiratory pressure (PEEP) of 14 cm H_2_O, and paralysis was initiated to support the patient's respiratory status. There was concern for a possible SARS-CoV-2 infection, so the patient was started on remdesivir. However, testing, including respiratory pathogen panel and bronchoalveolar lavage, showed no evidence of SARS-CoV-2 infection, so remdesivir was stopped.

Despite this, the patient continued to have poor oxygenation which prompted the decision to start the patient on ECMO. Sweep gas flow was set to 2 L/min with 100% FiO_2_. Two days after initiation of ECMO, the patient started to show improvement in oxygenation, and FiO_2_ was weaned successfully to 20%. On Hospital Day 9, the patient was decannulated from ECMO. The next day, as the patient's oxygenation continued to show improvement, he was extubated.

The investigations into the etiology of these events showed no evidence of respiratory infection, heart failure, or hemorrhage. The only pertinent discovery was a previous history of latent tuberculosis (TB) that had been treated approximately 10 years prior to admission, so the patient was started on isoniazid with vitamin B_6_ out of an abundance of caution by the infectious disease consulting team. The patient continued to improve with observation and supportive management. On Postop Day 19, the patient was discharged from the hospital with an antirejection regimen of tacrolimus, mycophenolate, prednisone, and valacyclovir and trimethoprim–sulfamethoxazole for infection prophylaxis.

### 2.2. Case 2

A male patient in his late 20s presented for renal transplantation. The patient was diagnosed with ESRD secondary to IgA nephropathy and was currently on peritoneal dialysis. The patient underwent deceased donor renal transplantation and received 20 mg basiliximab as part of the acute organ rejection induction protocol. On Postop Day 3, the patient received his second dose of basiliximab 20 mg. He also received a routine blood transfusion with 1 unit of packed red blood cells (pRBC) for a hemoglobin of 6.7 g/dL. Later that same day, the patient showed signs of hypothermia and went into cardiac arrest with pulseless electrical activity (PEA). The patient underwent acute cardiac lifesaving (ACLS) protocol and return of spontaneous circulation (ROSC) was achieved. However, during the intubation procedure, the patient became pulseless once more, and ACLS protocol was reinitiated. The patient received seven doses of epinephrine and calcium carbonate before ROSC was achieved again. Point-of-care transthoracic ECHO showed a hyperdynamic heart that was underfilled without any evidence of lung disease. Despite the repeat doses of epinephrine, the patient's blood pressure continued to be low. Bedside lab results showed respiratory acidosis and anemia with hemoglobin of 7.1 g/dL. The patient was given 3 units of pRBCs and 1 unit of plasma. Repeat hemoglobin measurement showed 10 g/dL, which was an appropriate response. However, blood pressure was low despite antihypotensive agents. Given this, along with the results of the ECHO, the cause of arrest was hypothesized to be due to hypovolemic shock along with a suspected aspiration event. This led to respiratory acidosis causing hyperkalemia and subsequent PEA arrest.

**Figure 1:**
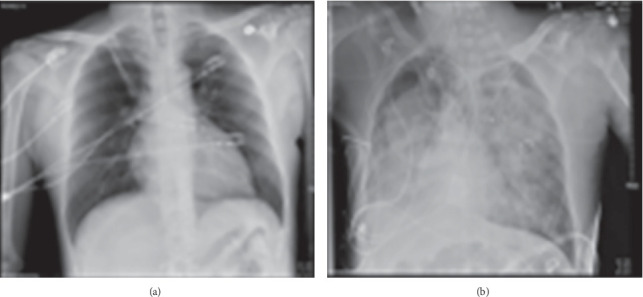
Chest X-rays demonstrating the rapid development of ARDS in Case 2. (a) Chest X-ray taken immediately after the patient underwent surgery demonstrating healthy lungs without evidence of infection. (b) On Postoperative Day 3, chest X-ray was consistent with ARDS.

Given the hypovolemia, a slow venous bleed was suspected, and the patient was taken to the operating room for an exploratory laparotomy where a perinephric hematoma was identified and evacuated. However, a source of active bleeding was not identified. The patient was returned to the ICU and placed on three separate antihypotensive agents along with fluid resuscitation with lactated ringers. The patient's respiratory status continued to deteriorate. Chest X-ray showed significant infiltrates, and a concern for ARDS was raised (Figure 1). The PEEP was increased from 7 to 12 cm H_2_O without significant improvement. A bedside bronchoscopy showed no evidence of mucus plugging. Samples were collected from a bronchoscopic alveolar lavage that showed presence of histoplasmosis and aspergillus, so antifungal agents as well as antibiotics were started for empiric coverage. Despite these adjustments, the patient did not show evidence of improvement. Therefore, the patient was started on venovenous ECMO. The flow was set to 1.35 L/min, with pump rate of 2370 rpm. Sweep gas flow was set to 2 L/min with 100% FiO_2_.

While on ECMO, there was trouble maintaining the flow rates in the setting of abdominal distention concerning for abdominal compartment syndrome. CT scan of the abdomen and pelvis showed an enlarging pelvic hematoma leading to a repeat surgical procedure for further exploration. Two arterial bleeds were identified and oversewn. A renal vein thrombus was also identified around the region of the anastomosis and addressed. The ECMO cannulas were also adjusted from the groin to the internal jugular vein to reduce limitations related to abdominal pressures. Despite this change in cannula placement, the treatment team continued to have trouble with low flow rates on ECMO. The patient returned to the operating room once more for a second reconfiguration, this time to place an Avalon cannula in the hopes of improving flow rates. After this surgery, the patient's ECMO flows improved to 2.5 L/min. Sweep continued to be adjusted, and FiO_2_ remained at 100%. The patient's progress was monitored via imaging. Chest X-rays showed improvement in the lungs with a progressive decrease in severity of infiltrates observed. On Hospital Day 12, 7 days after ECMO initiation, the patient was weaned down to 21% FiO_2_ and decannulated. Soon after, the patient was extubated as well. The patient continued to show improvement and was eventually discharged on Hospital Day 23.

## 3. Discussion

The presence of complications after renal transplantation is well studied. Typically, most surgical complications are vascular in nature such as thrombosis or stenosis of the renal artery and/or vein [5]. Other common complications are typically urologic or nephrogenic in nature [6]. Nonsurgical complications are typically driven by patient comorbidities, such as cardiovascular disease and diabetes. Also, immunosuppressive therapies can cause drug-related toxicity and increase the risk of infections in these patients [7]. The lungs have been determined to be the most common location for infections in this population [8]. A study of nine transplant centers showed that approximately 3% of renal transplant patients are admitted to the ICU for acute respiratory failure. Among these patients, the primary cause of respiratory failure is bacterial infection followed by cardiogenic edema and finally, extrapulmonary bacterial sepsis resulting in ARDS [9]. However, none of these patients required ECMO to support oxygenation.

In this report, we present two cases of patients without severe comorbidities or immunosuppression prior to transplant who experienced acute respiratory failure on Postop Day 3 after renal transplantation requiring ECMO. In the first case, the patient had no risk factors for respiratory decline apart from possible pulmonary edema seen as infiltrates on imaging. However, the rapid decline that the patient experienced without evidence of systemic fluid overload or reduced cardiac ejection fraction cannot be easily explained by pulmonary edema alone. Furthermore, the patient's infectious workup was benign aside from a remote history of latent TB infection that had been previously treated.

The second case is more complicated. The patient had multiple risk factors for the development of respiratory distress. The patient received a transfusion about 5 h before the onset of symptoms, making a transfusion-related acute lung injury (TRALI) possible. The patient was examined for evidence of ABO and Rh incompatibility, and lab results including a direct antiglobulin test were negative. However, the transfused product was not tested for antibodies due to lack of a sample, so TRALI could not be completely ruled out. Nonetheless, the timing of symptom onset is important as TRALI typically presents within minutes after transfusion, with most presenting within the first 4 h, supporting the hypothesis that TRALI was not the cause of the patient's rapid decline.

Another possible cause of respiratory failure is the patient's positive infectious disease studies indicating fungal growth that does present a source of infection that could have resulted in pneumonia. However, the patient did not meet the criteria for sepsis or have indications of severe infection such as leukopenia or temperature changes. While infection could have played a role, the rapid onset and severity of the patient's symptoms point to an additional and/or separate etiology. A final possible cause is aspiration-related respiratory failure. Before symptoms developed, the patient had a coughing episode with drinking fluids. However, the symptoms were not persistent, and the patient was stable immediately after the episode, making aspiration a less likely precipitating event. Nevertheless, aspiration pneumonitis could be a contributing factor for the events that occurred.

Although the second patient had several risk factors and possible other etiologies for his acute respiratory failure, the timing of the onset of symptoms and both patients' hospital courses had many similarities: Both patients started to decline after receiving their second dose of basiliximab, and despite maximal ventilator settings, both patients were unable to properly oxygenate without the assistance of ECMO. This demonstrates that there may have been a common inciting factor in both patients' courses that caused this complication.

Several different immunosuppressive classes have been used as part of the acute rejection prevention protocol for renal transplant. Different agents are used as part of the induction and the maintenance phases, with steroids often used in both phases. Common induction agents are rabbit antithymocyte globulin (rATG) and IL-2 receptor inhibitors such as basiliximab. These agents are typically used in conjunction with corticosteroids. Research thus far has shown that rATG has a known association with ARDS, with some studies showing that patients experiencing graft failure are at increased risk of developing this complication [10]. However, this study was conducted prior to the widespread use of basiliximab as an induction agent.

A 2022 study compared the prevalence of COVID-19 and associated ARDS in renal transplant patients receiving either thymoglobulin or basiliximab [11]. Their results showed that older patients had significantly higher rates of ARDS and mortality when receiving thymoglobulin compared to basiliximab. However, in younger patients, the risk of developing ARDS was higher in patients receiving basiliximab versus thymoglobulin. Mortality in younger patients who developed ARDS within the first 3 months postrenal transplantation was double in the group receiving basiliximab compared to those receiving thymoglobulin.

Additionally, there are several case reports of patients developing noncardiogenic pulmonary edema after renal transplantation and induction with basiliximab [12]. However, these patients did not require ECMO. Given this association between basiliximab and pulmonary complications, it is reasonable to hypothesize that this induction agent played a role in these patients' rapid development of respiratory failure. Importantly, the reports thus far raise the concern that young patients are especially at risk for this complication.

## 4. Conclusion

Basiliximab may play a role in the development of acute respiratory failure after renal transplantation in young patients. Clinicians who use this agent as part of induction therapy in renal transplantation should be aware of this potential complication and observe younger patients closely for signs of respiratory failure. Furthermore, it would be reasonable to target future studies into examining the effects of basiliximab on the respiratory system of young patients.

## Data Availability

The data that support the findings of this report are available on request from the corresponding author. The data is not made publicly available due to privacy and ethical restrictions.
